# Methodological Insights from Low-Vacuum SEM for Morphological Analysis of Schwann Cells on Electrospun Scaffolds

**DOI:** 10.3390/polym18111407

**Published:** 2026-06-05

**Authors:** Paulina Salazar-Aguilar, Andrea Barrenechea Sánchez, Karina Godoy Sánchez, Paulina Martínez-Rodríguez, Dimitrius Leonardo Pitol, María Eugenia González-Quijón, Fernando José Dias

**Affiliations:** 1 Doctoral Program in Dental Sciences, Dental School, Universidad de La Frontera, Temuco 4780000, Chile; p.salazar10@ufromail.cl; 2 Dentistry Program, Faculty of Medicine and Health Sciences, Universidad Mayor, Temuco 4780000, Chile; 3 Master Program in Dentistry, Dental School, Universidad de La Frontera, Temuco 4780000, Chile; a.barrenechea01@ufromail.cl; 4 Scientific and Technological Bioresource Nucleus (BIOREN), Universidad de La Frontera, Temuco 4780000, Chile; karina.godoy@ufrontera.cl; 5 Doctoral Program in Applied Cellular and Molecular Biology, Universidad de La Frontera, Temuco 4780000, Chile; paulinaconstanza.martinez@ufrontera.cl; 6 Department of Oral and Basic Biology, School of Dentistry of Ribeirao Preto, University of Sao Paulo, Ribeirao Preto 14040-904, Brazil; dimitrius@forp.usp.br; 7 Department of Chemical Engineering, Universidad de La Frontera, Temuco 4780000, Chile; mariaeugenia.gonzalez@ufrontera.cl; 8 Department of Integral Adult Dentistry, Oral Biology Research Centre (CIBO-UFRO), Dental School, Facultad de Odontología, Universidad de La Frontera, Temuco 478000, Chile

**Keywords:** glial cells, SEM, nerve regeneration

## Abstract

Schwann cells (SCs) are critical effectors of peripheral nerve regeneration, and their interaction with biomaterial scaffolds is a key parameter in neural tissue engineering. This pilot study described and evaluated protocols for a morphological, quantitative, and morphometric analysis of SCs seeded on electrospun polyhydroxybutyrate (PHB) scaffolds using variable-pressure scanning electron microscopy (VP-SEM) under a low vacuum, without a metal coating. Six protocols were compared, varying the number of seeded cells (50,000 or 100,000) and the method used to label the seeded face of the scaffold: no marking, graphite pencil, or permanent ink (Sharpie). Confocal microscopy confirmed SC viability and adhesion. The VP-SEM analysis revealed that seeding 100,000 cells significantly increased the number of detectable cells on the scaffold surface. Graphite labeling was associated with higher cell counts and a more stellate morphology, consistent with the biocompatibility of carbon-based materials reported in the literature. Conversely, ink labeling appeared to inhibit SC adhesion. A refined protocol for measuring SC extensions using ImageJ’s ROI Manager and segmented line tools was also established. These findings provide practical methodological insights to improve the reliability and reproducibility of SC morphological analyses on ultra-thin polymeric scaffolds, with implications for peripheral nerve regeneration research.

## 1. Introduction

Schwann cells constitute the principal glial cells of the peripheral nervous system, fulfilling essential roles in axonal support, myelination, and maintenance of the peripheral neural microenvironment [1,2]. Broadly, myelinating Schwann cells form compact myelin around larger axons, whereas non-myelinating Schwann cells, such as Remak cells, envelop small-caliber axons and provide trophic support without generating compact myelin [1,3]. This functional diversity demonstrates that Schwann cells serve not only as structural components but also as active regulators of nerve homeostasis, axon–glial stability, and peripheral nerve tissue organization [1,4].

The significance of Schwann cells becomes particularly evident following nerve injury, as a substantial portion of the peripheral nervous system’s regenerative capacity relies on their phenotypic plasticity [2,5]. After axonal damage, Schwann cells can be reprogrammed into a reparative phenotype characterized by controlled demyelination, myelinophagy, secretion of neurotrophic factors, remodeling of the extracellular matrix, and formation of Büngner bands that guide axonal regrowth [3,5,6]. As a result, Schwann cells represent a critical biological target in nerve tissue engineering, as their response to biomaterials can influence the creation of a reparative niche conducive to peripheral regeneration [2,4].

Schwann cell biology is intimately associated with demyelinating diseases and disruptions of axon–glial units [7]. In hereditary neuropathies, numerous defects involve genes expressed by myelinating Schwann cells, whereas in acquired neuropathies, such as Guillain-Barré syndrome or chronic inflammatory demyelinating polyneuropathy, immune-mediated damage impairs the formation and maintenance of peripheral myelin [7]. Furthermore, in diabetic peripheral neuropathy, altered Schwann cell plasticity is proposed to contribute to demyelination, diminished regenerative capacity, and progressive peripheral nerve deterioration [6]. From a biomaterial perspective, this context is significant because the adhesion, expansion, and morphology of Schwann cells on a surface can provide early indicators of functional biological compatibility, extending beyond a material’s physicochemical stability [3,4].

Within this framework, assessing early adhesion and surface-associated cell morphology are essential for evaluating biomaterials designed for neuroregeneration [3,8]. Parameters, such as cell spreading, elongation, substrate contact continuity, and the presence of filopodia and lamellipodia, provide insights into whether a surface delivers the topographic and physicochemical cues that support favorable cell–material interactions [4,8]. For Schwann cells, this information is particularly valuable, as their morphology and spatial organization are closely associated with the biological states relevant to axonal support, migration, and nerve repair [2,5].

Scanning electron microscopy (SEM) is particularly advantageous for this application, as it enables high-resolution visualization of biomaterial’s surface topography, cell morphology, anchor points, and the physical interface between the cells and substrate [9]. In contrast to conventional light microscopy, SEM provides a more precise assessment of cell attachment and surface extension, as well as detailed characterization of membrane features and interactions with the micro- and nanotopographies of a biomaterial [9,10]. However, the analytical value of SEM is highly dependent on the preparation protocol, as fixation, dehydration, drying, assembly, and acquisition conditions can induce shrinkage, structural collapse, material loss, and charging artifacts that may distort the morphological interpretation [9,10].

In this context, SEM modalities that employ variable pressure and vacuum conditions are particularly valuable for analyzing delicate biological samples and fragile biomaterials. Variable-pressure SEM (VP-SEM) operates at low pressure rather than high vacuum, enabling high-resolution imaging with minimal sample preparation, thereby reducing the time, cost, and sample damage [11]. While this technique has been utilized in other biological systems, its methodological advantages are especially pertinent for studying Schwann cells on thin substrates, where an aggressive preparation can alter cell morphology and the cell–material interface. Additionally, low-vacuum SEM (LV-SEM) has been applied to peripheral nerve analysis, facilitating rapid examination of tissue microarchitecture and myelin using backscattered electrons, and providing consistent measurements and higher resolution than light microscopy without the need for metal coating of the resin block [12]. Collectively, these approaches underscore the importance of selecting SEM modalities that optimally preserve the true morphology of cells and their interactions with biomaterials.

These considerations become even more critical when the biomaterial is extremely thin, flexible, or brittle, as handling during cultivation and processing can compromise its original geometry and spatial orientation [9,13]. In ultra-thin membranes, the combination of nanoscale dimensions, high porosity, and low rigidity can enhance certain biomimetic properties but also increase the experimental complexity associated with their management, support, and morphological evaluation [13]. In cell culture scenarios, these characteristics can hinder accurate identification of the face on which cells are seeded, particularly if the material shifts, curves, or inverts during prolonged exposure to the medium or during pre-processing for SEM [9,13]. If the analyzed face does not correspond to the seeded side, interpretation of adhesion and cellular compatibility may be methodologically biased, even if the image quality appears adequate [3,9]. Therefore, for ultra-thin biomaterials, the implementation of seed-side marking and orientation strategies is a critical methodological consideration [3,9].

In this context, the comparison of graphite and ink serves as an evaluation of labeling methods that enable more reliable preservation and identification of the seeded side, without interfering with the analysis surface or the morphological interpretation of cell adhesion [9,10]. This methodological requirement reflects a broader challenge in the field, as comparisons among studies on biomaterials and nerve regeneration are often constrained by protocol diversity, differences in evaluation methods, and a lack of standardization at critical experimental stages [3].

Therefore, the objective of this pilot study was to establish and discuss insights into the optimal strategy for SEM observation of Schwann cell adhesion on ultra-thin polymeric biomaterials, specifically identifying whether graphite or ink labeling provides more reliable identification of the seeded face and enables a more robust evaluation of the morphological compatibility between a polymeric electrospun biomaterial and Schwann cells.

## 2. Materials and Methods

The biosafety aspects of the present in vitro study protocol were approved by the Ethical–Scientific Committee of the Universidad de La Frontera on September, 2025, with approval number 120_25.

### 2.1. PHB Scaffold Fabrication and Preparation for Cell Culture

**Figure 1:**
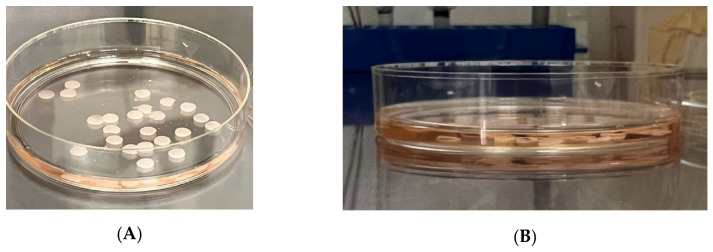
Preparation of PHB samples for cell culture. (**A**) Laminar scaffolds of PHB cut into a circular shape with a diameter of 5 mm, immersed in cell culture medium. (**B**) PHB scaffolds are soaked in culture medium at the bottom of the petri dish.

The preparation of the polyhydroxybutyrate (PHB) scaffold was performed as previously described in [14]. Commercial PHB (poly[(R)-3-hydroxybutyric acid], Sigma Aldrich, St. Louis, MO, USA) at a concentration of 10% was dissolved in chloroform at 60 °C for 24 h. The manufacturing parameters were a voltage of 25 kV, a flow rate of 1 mL/h, a distance between the collector and needle of 15 cm, a rotation speed of 50 rpm, and a collection time of 150 min [14]. Once prepared, the samples were cut into a round shape with a 5 mm diameter using an office paper punch under sterile conditions (Figure 1A). This size was chosen because it is ideal for accommodating the PHB scaffold at the bottom of the wells in 96-well cell culture plates. After cutting the samples from the PHB scaffolds, it was necessary to submerge them in cell culture medium for 24 h at 4 °C to allow them to absorb the medium and sink into the wells of the culture plate (Figure 1B).

### 2.2. Cell Culture—Schwann Cells

The Schwann cell culture was performed as previously described in [15,16]. Commercial nerve Schwann cells acquired from Merck-Sigma (Burlington, MA, USA) (SCL 4.1/F7, catalog no. 93031204), from the “ECACC—European Collection of Authenticated Cell Cultures,” were used. The Schwann cells were thawed and then expanded in cell culture containers to achieve at least 70% confluence. The cells killed during expansion were discarded by washing the container.

The live cells were detached from the container by trypsin, quantified using a Neubauer chamber, and then suspended in volumes with concentrations previously determined to achieve the quantities proposed in the present study for use in culture plates, with the base covered by scaffolds of polyhydroxybutyrate (PHB) microfilaments fabricated using the electrospinning method in [14] and prepared as previously described.

Schwann cells supplemented in a solution of Ham’s F-12 culture medium, 1% glutamine, 10% Fetal Bovine Serum (FBS), and 1% Penicillin–Streptomycin at 37 °C in a humidified atmosphere at 5% CO_2_ were monitored and maintained in a cell culture chamber on round well-cut PHB laminar scaffolds in a plate of 96 wells [14,15,16].

### 2.3. Labeling and Cell Amounts Analysis Protocols—Study Groups

Due to the difficulties inherent to the technique of identifying the side of a laminar PHB scaffold seeded with cells, in addition to the various exchanges of liquids in the cell culture that can easily turn the sample, an analysis of different methods of labeling the seeded side and with different quantities of seeded cells (5 × 10^4^ or 10 × 10^4^ cells) was performed.

Thus, the unmarked samples were called the controls; there were samples marked with a graphite pencil and, finally, samples marked with office paint (Sharpie ink). To facilitate understanding of the groups analyzed, we describe the protocols in the present study in Table 1.

### 2.4. VP-SEM Processing and Quantification of Cells and Measurements of Cell Extensions

The Schwann cells were evaluated after 3 days of incubation in the different groups to assess their adherence and morphological and morphometrical characteristics.

For this purpose, the cells were fixed in 2.5% glutaraldehyde (EMS, USA) in Sorensen’s phosphate buffer 0.1 M (Electron Microscopy Science, Hatfield, PA, USA) for 30 min, washed in ultrapure water, and taken to the Hitachi SU3500 scanning electron microscope (Hitachi, Tokyo, Japan) of the BIOREN-UFRO [14,15,16].

**Figure 2:**
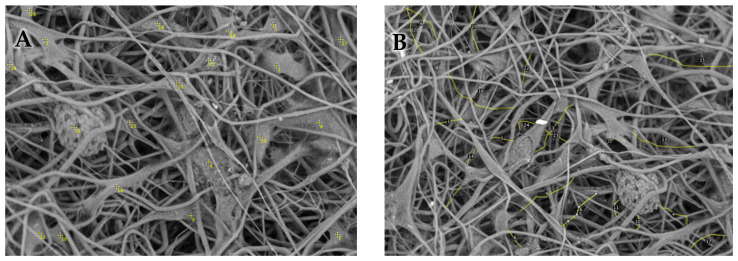
Quantitative and morphometric analysis of the VP-SEM using ImageJ software. (**A**) Quantification of the number of cells using the “Multi-Point” tool. (**B**) Measurements of the extensions using the “segmented line” tool systematized by the “ROI manager” tool. Mag: ×500.

For the morphometric and quantitative analysis of SCs, VP-SEM photomicrographs at a standard magnification of ×500 were used. Using ImageJ software (Version 1.54 g—National Institutes of Health—NIH, Bethesda, MD, USA) for the analysis of the number of cells, the “multi-point” tool was employed (Figure 2A); the values of the quantities were recorded in spreadsheets and later compared among the different groups of the study.

For the analysis of cell extensions, the photo scale was initially standardized (Analyze > Set Scale) using the tool to trace a line on the scale in the microscope images. The extensions were measured with the help of the “segmented line” feature together with the “ROI manager” tool to help control and identify the measured extensions and the recorded measurements (Figure 2B). The values obtained from the cell extension measurements were stored in the form of a spreadsheet and later compared among the different groups.

### 2.5. Confocal Microscopy Analysis

Following the VP-SEM analysis, the SCs were stained with 1 µM DAPI (Thermo Fisher, Waltham, MA, USA) [17] in the dark for 10–15 min, then washed three times with PBS. Protein labeling was followed with 1% *w*/*v* Fastgreen (Merck, Rahway, NJ, USA) [18] in PBS for 5–10 min, with three subsequent washes. The sample was imaged on a Fluodish plate using confocal microscopy (Olympus FV1000—Tokyo, Japan; Mag: X60, water immersion, X2 zoom) at 405/450 nm and 633/700 nm. This analysis assessed the SCs and PHB scaffold integrity and the approach’s feasibility following the VP-SEM analysis.

### 2.6. Data Analysis

Initially, the normality of the cell quantification data and the prolongation measurements were analyzed by a Shapiro-Wilk test. The data from the 6 protocols that followed a normal distribution were analyzed using a one-way analysis of variance (ANOVA) with Bonferroni’s post hoc test. The data that were not normally distributed were analyzed using a Kruskal–Wallis test with Dun’s post hoc test. In both cases, the significance level was *p* = 0.05.

## 3. Results

### 3.1. Morphological Analysis (VP-SEM) of Schwann Cells Seeded on the PHB Scaffold

#### 3.1.1. Control 50 and Control 100 Protocols

**Figure 3:**
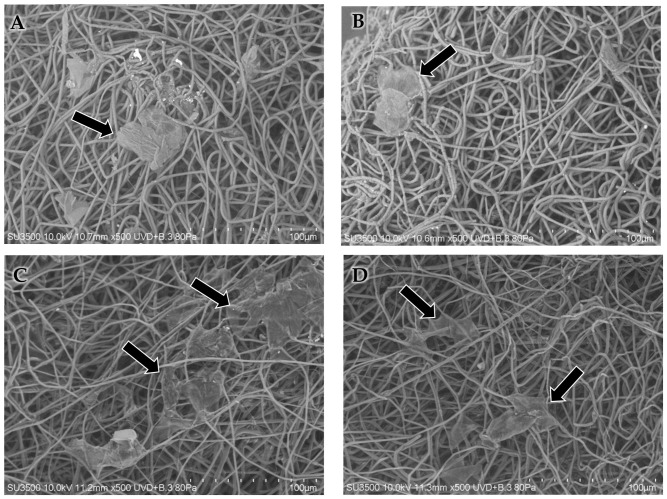
(**A**,**B**) Control 50—The presence of a few Schwann cells (arrows) is observed. (**C**,**D**) Control 100—A greater number of Schwann cells (arrows) with more evident cell extensions are observed. Mag: ×500.

The images obtained by the VP-SEM for the control protocol show Schwann cells adhered to the PHB scaffold’s electrospun fibers. In general, the cells have a flatter shape and are superimposed on the PHB fibers, with a visually evident greater number of cells observed in the Control 100 group compared to Control 50, as well as a greater presence of cell extensions in the Control 100 group (Figure 3).

#### 3.1.2. Graphite 50 and Graphite 100 Protocols

**Figure 4:**
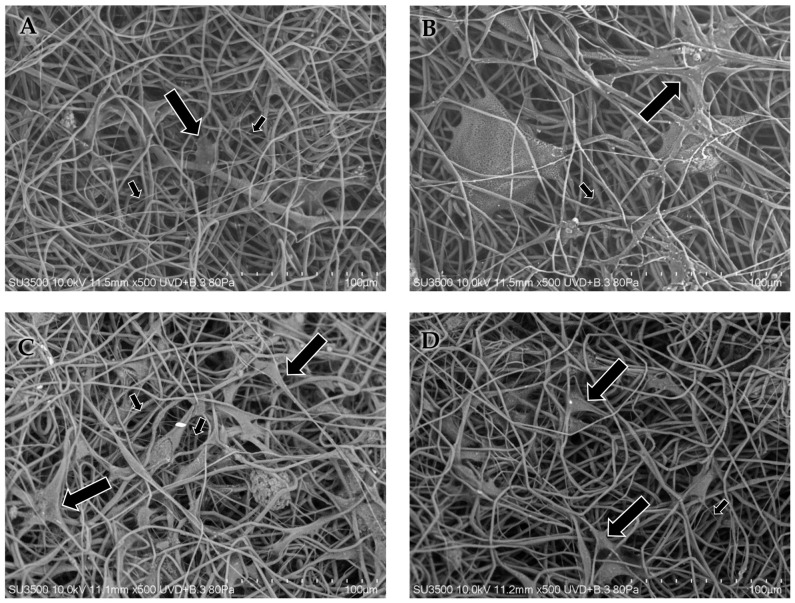
(**A**,**B**) Graphite 50—The presence of Schwann cells (arrows) with a star shape and many cell extensions (short arrows) are observed. (**C**,**D**) Graphite 100—The presence of many Schwann cells adhered in a starry shape (arrows) is observed; however, a smaller number of cell extensions are observed. Mag: ×500.

The photomicrographs of the samples prepared using the graphite protocol reveal Schwann cells adhered to the scaffold fibers, as in the control protocol. However, in this group, the cells are more stellate and interspersed with the PHB fibers. Again, the protocol that received the most graphite, the Graphite 100 cells, show a greater number of cells than the Graphite 50 protocol. However, between these protocols, the most evident presence of cell extensions is in the group with the fewest cells (Graphite 50), which is noteworthy (Figure 4).

#### 3.1.3. Ink 50 and Ink 100 Protocols

**Figure 5:**
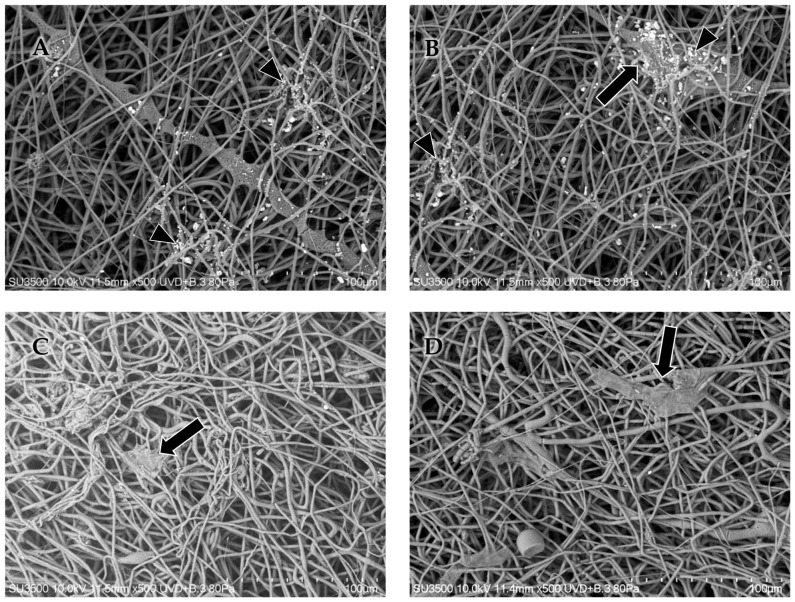
(**A**,**B**) Ink 50—Few adhered cells (arrows) and small electrodense structures (arrowheads) suggestive of traces of marking ink are observed. (**C**,**D**) Ink 100—Few cells (arrows) with a flatter appearance are observed. Mag: ×500.

The photomicrographs obtained from the protocol using ink showed little cell adhesion to the PHB scaffold. In the Ink 50 group, small electron-dense structures, possibly representing the ink used for scaffold marking, were observed, with a few cells adhering to the adjacent region. The Ink 100 samples had more cells than the Ink 50, but with flatter characteristics; the cells observed in the Control 50 and Control 100 groups were similar (Figure 5).

### 3.2. Quantification of the Number of Schwann Cells

**Figure 6:**
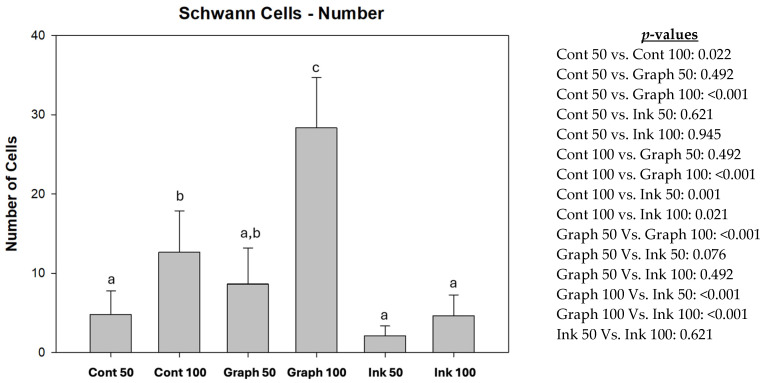
Graph of the preliminary analysis of the Schwann cell count detected in the study. Because the cell counting data follow a normal distribution, an ANOVA is used to compare the groups, and the graph shows the mean and standard deviation for each group. Equal letters indicate no significant differences (*p* < 0.05). The letter a indicates a non-significant difference (*p* > 0.05) between the Cont 50, Graph 50, Ink 50 and Ink 100 groups. The letter b indicates a significant difference (*p* < 0.05) compared to the Cont 50, Graph 100, Ink 50, and Ink 100 groups. The letter c indicates the significant difference (*p* < 0.001) of the Graph 100 group compared to all other groups in the study.

The preliminary analysis of the cell quantification performed revealed significant differences (*p* > 0.05) among the protocols performed in the present study, with the data showing a normal distribution. The Control 50 (4.8 ± 2.9 cells) and Graphite 50 (8.7 ± 4.5 cells) protocols had a significant number of cells detected (*p* < 0.05) compared to similar protocols with higher numbers of cells, the Control 100 (12.7 ± 5.2) and Graphite 100 (28.3 ± 6.3). Only the ink-labeled samples (ink 50, 2.2 ± 1.2 cells; Ink 100, 4.7 ± 2.6 cells) did not show this difference. The Graphite 100 labeling protocol resulted in a significantly higher number of cells (*p* < 0.001) across all study protocols (Figure 6).

### 3.3. Schwann Cell Extensions

The preliminary descriptive data for the measurements of the SC extensions are presented in Table 2.

**Figure 7:**
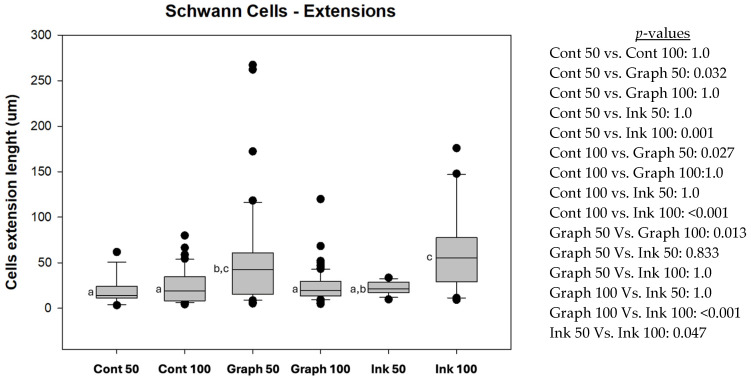
Graph of the preliminary analysis of the measurements of the detected cell extensions. Due to the non-normal distribution of cell extension data, a Kruskal–Wallis test is used to compare the different groups. Thus, the data are represented in the form of box plots with the distribution represented as the median, Q1—25% and Q3—75%. Similar letters indicate non-significant differences (*p* > 0.05). The letter a indicates that there are no significant differences among the Control 50, Control 100, Graphite 50, and Ink 50 groups. The letter b indicates a non-significant difference between the Graphite 50 and Ink 50 groups only. The letter c indicates that there are no significant differences between Graphite 50 and Ink 100.

Analysis of measurements of the detected Schwann cell extensions reveals differences across the protocols used in the study. The data do not present with a normal distribution, so their graphic representation is a boxplot. The distribution of the measurements shows smaller extensions and no statistical difference (*p* > 0.05) among the Control 50, Control 100, Graphite 100, and Ink 50 protocols. The Graph 50 and Ink 100 protocols stand out for having significantly higher measurements (*p* < 0.05) than the other groups (Figure 7).

### 3.4. Analysis of Schwann Cells Seeded with PHB Scaffold After VP-SEM Analysis—Confocal Microscope

**Figure 8:**
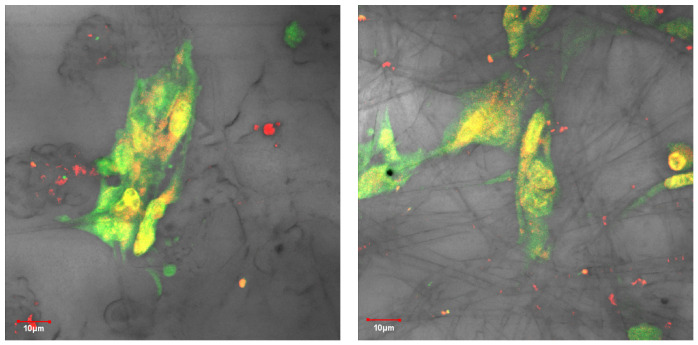
PHB scaffold samples with adhered and viable Schwann cells.

The confocal microscopy images acquired from the same samples following the VP-SEM analysis demonstrate the interaction and morphology of Schwann cells fixed with the PHB electrospun scaffold (Figure 8). Although the primary focus of the study is the VP-SEM analysis, the image processing and acquisition methods employed indicate the potential for additional analyses of the same sample preparations using various microscopy techniques.

## 4. Discussion

This in vitro pilot study describes the protocol and provides insights for processing and conducting morphological, quantitative, and morphometric analyses of SCs seeded on a polyhydroxybutyrate (PHB) electrospun scaffold, using variable-pressure scanning electron microscopy (VP-SEM) under low-vacuum conditions. The primary differences among the protocols evaluated are the number of seeded cells (50,000 or 100,000) and the method used to mark the seeded side: no marking, graphite marking, or common ink marking.

The present study introduces several methodological contributions to the analysis of Schwann cells seeded on electrospun polymeric scaffolds. Unlike previous studies that relied on metal-coated SEM, we employ VP-SEM at low vacuum without coating, preserving finer details of the cell–fiber interface. A systematic comparison of scaffold face-labeling strategies addresses the underreported problem of sample inversion during processing, a critical source of methodological bias in ultra-thin membranes. The cell extension measurements are refined using the combined “segmented line” and ROI Manager tools in ImageJ, enabling precise, non-redundant morphometric analysis of structures that are visually similar to PHB fibers. Additionally, sequential analysis of the same specimen by VP-SEM followed by confocal microscopy demonstrates the feasibility of multimodal characterization from a single sample preparation. Together, these methodological elements offer a more reproducible and informative framework for evaluating cell–biomaterial interactions in peripheral nerve regeneration research.

The characterization of the PHB scaffold used in the present study was previously reported and discussed in a study by our research group [14], which explored the behavior of SCs across different PHB scaffold manufacturing configurations. In that study, it was found that the cells showed greater viability when the pores/spaces between the electrospun fibers were larger, and the thickness of these fibers was greater [14]. The manufacturing parameters chosen in the present study follow the recommendations of the previous study to achieve the best PHB scaffold characteristics and the highest cell viability.

Previous studies analyzing SCs seeded on electrospun polymeric scaffolds composed of different biomaterials [14,16,19,20,21,22,23,24,25,26,27,28,29] have primarily examined their morphology, cellular interactions, and adhesion using scanning electron microscopy (SEM), with sample coatings such as gold [19,21,22,24,25], gold–palladium [20], or platinum–gold [26]. Coating samples enables them to withstand a higher SEM energy (kV) without structural damage and allows for greater magnification. However, this process may obscure important details of cellular interactions with scaffold fibers. Notably, only studies conducted by this research group [14,15,16] have utilized VP-SEM without metal coating, thereby revealing finer details of SC interactions with PHB scaffold fibers.

Regarding the optimal number of cells for sowing, our results indicate that, for both the control and graphite-labeled groups, the number of cells detected as interacting with the PHB scaffold were significantly higher when the largest number (1 × 10^5^) was sown.

Previous studies that seeded SCs onto electrospun polymeric scaffolds have reported substantial variability in the total number of cells used, ranging from 50 cells/mm^2^ to 5000, 12,000, 15,000, 20,000, 25,000, 50,000, and 100,000 cells [14,16,19,20,21,22,23,24,25,26,27,28,29]. Without a clear indication of prior protocols and given that the main purpose of our VP-SEM morphological analysis was to examine the cellular interactions with the material under study, we propose that it is more advantageous to use a protocol with a greater number of cells rather than fewer.

Marking the PHB laminar scaffold face where the SCs were seeded was found necessary in previous studies conducted by this research group [14,16]. Once cells are seeded on one side of a scaffold, subsequent procedures, such as changing the culture medium and performing baths with fixatives and buffers during cell processing, can easily rotate the sample by 180 degrees. As a result, during VP-SEM analysis, it may become unclear which scaffold face was originally seeded with cells.

Previous studies have not mentioned the need to mark the scaffold face that is seeded. Therefore, only the results from the present study regarding the three proposed marking methods are discussed.

Our results suggest that marking with common workshop paint (Sharpie) inhibited SCs, whereas the graphite marking appeared to stimulate cell proliferation and interaction with the material. In the graphite-marked samples, a greater number of cells with a more stellate morphology were observed interspersed among the PHB fibers.

While acknowledging the lack of direct evidence of cell proliferation and viability, the analysis of the results was based solely on the cell counts observed using the VP-SEM analysis method. These results are consistent with previous studies demonstrating that carbon-based compounds, such as graphene [25] and multiwalled carbon nanotubes [28], also produce positive effects on SCs.

Another key objective of this study was to develop a protocol for analyzing SC extensions, building upon the methodologies previously established by Quidel-Necul et al. [16]. In the current work, the measurement process was refined by applying the “ROI manager” tool in conjunction with the “segmented line” feature of ImageJ. This approach enabled the precise marking and identification of measured structures within the photomicrographs, thereby preventing repeated measurements of the same structure.

The presence of SC extensions offers significant insight into the mechanisms underlying cellular network development on electrospun scaffolds [16]. Following peripheral nerve injury, SCs initiate a repair program marked by pronounced elongation and extensive branching, resulting in long, parallel-oriented extensions that facilitate axonal regeneration [30]. The formation of these cell extensions is dependent on reciprocal interactions between axons and SCs, mediated by extrinsic signals from both axons and the extracellular matrix. These signals collectively determine SCs’ fate and drive the cytoplasmic reorganization required for extension formation [31]. Axon-derived molecules, such as Neuregulin 1 (NRG1), bind to SC receptors and activate the signaling pathways that coordinate cellular behavior and promote the formation of these extensions [32].

Previous studies that evaluated the interaction of Schwann cells (SCs) with electrospun scaffolds have not reported or analyzed cellular extensions. This omission may have resulted from the loss of morphological detail caused by sample coating. Instead, a few of these studies have focused on neurite growth [26,29], which are cellular extensions exclusive to neurons.

Overall, the measurements of cell extensions indicate that the greatest prolongations occurred in the groups with the fewest detected cells (Graphite 50 and Ink 100). This relationship suggests that SCs seeded on a scaffold may form networks and, when fewer viable cells are present, extend longer processes to reach more distant cells. However, this interpretation, based on a preliminary analysis, requires confirmation by future studies employing more robust methodologies.

The primary advantage of the described analysis protocol is the simplicity of the sample preparation process. SCs are seeded onto a laminated scaffold, which is cut to fit the bottom of the wells in a 96-well plate (approximately 5 mm in diameter) using a sterile paper punch. Maintenance of the seeded cells is achieved by regularly changing the culture medium. For analysis by variable-pressure scanning electron microscopy (VP-SEM), fixation is performed one day prior with 2.5% glutaraldehyde, without the use of additional post-fixation agents or metal coatings. The resulting images reveal detailed interactions between the cells and the electrospun polymeric scaffold, enabling assessment of cell adhesion, morphology, and quantification of these cells.

The analysis conducted using a confocal microscope on the samples previously examined with VP-SEM demonstrates the feasibility of performing multiple microscopic analyses on a single sample. This approach could reduce the required sample preparation and consequently lower the overall costs associated with cell analyses.

The primary challenges of this method include the potential for the seeded sample to rotate 180 degrees if the seeded side is not clearly identified. Additionally, analysis of cell extensions is complicated by their morphological similarity to the electrospun polyhydroxybutyrate (PHB) scaffold fibers. Both cells and their extensions may also penetrate the porous structure of the PHB scaffold, hindering accurate analysis of these features. These limitations may be addressed by improving observer calibration regarding distinguishing between PHB fibers and cell extensions. Furthermore, future implementation of artificial intelligence systems could enhance measurement accuracy and reduce human error.

Overall, the cellular quantification method demonstrates reduced susceptibility to confounding factors and can be readily applied to studies investigating the interaction of SCs with scaffolds of similar characteristics. Further studies are required to more comprehensively analyze the use of the same sample for an initial examination with VP-SEM and a subsequent one with a confocal microscope. Nevertheless, the preliminary results of this pilot study demonstrate the feasibility of this approach.

The main limitation of the study is the non-inclusion of methods for cell proliferation/viability and migration analysis. Although this was not the focus of the study, including this analysis would certainly improve the interpretation of the observed cellular behavior by comparing the different methods of marking the face of the scaffold seeded with the cells. Thus, the authors state that the results of this pilot study are mainly descriptive and that, in the future, they should be functionally validated using the methods mentioned above.

## 5. Conclusions

The present pilot study provides insights into precautions regarding sample preparation and morphological analyses of Schwann cell interactions with electrospun polymeric scaffolds using variable-pressure scanning electron microscopy (VP-SEM), in comparison to conventional scanning electron microscopy (SEM), which requires sample coating.

The preliminary descriptive results suggest that seeding a higher number of cells enhances the likelihood of successful localization and analysis on an electrospun polymeric scaffold. Additionally, marking the cell-seeded side is a methodological consideration that reduces the risk of confusion during SEM morphological analysis. The analysis of SC extensions is essential, as it provides valuable insights into their behavior when using the described methodology.

The analyses and discussion of methodological advantages presented in this study are preliminary and mainly descriptive; future constructive discussions are encouraged to further refine the morphological analysis of SCs seeded on polymeric scaffolds. For example, the functional confirmation of cell quantification using the appropriate cellular proliferation and viability methods.

This work represents an initial step toward more complex and sophisticated analyses, which will contribute to a deeper understanding of SC behavior and their applications in peripheral nerve regeneration.

## Figures and Tables

**Table 1:** Schwann cell seeding amounts and seeded side labeling protocols.

Protocol	Number of Cells Sown	Type of Marking
Control 50	5 × 10^4^ cells	No labeling
Control 100	10 × 10^4^ cells	No labeling
Graphite 50	5 × 10^4^ cells	Graphite pencil
Graphite 100	10 × 10^4^ cells	Graphite pencil
Ink 50	5 × 10^4^ cells	Ink pencil (Sharpie)
Ink 100	10 × 10^4^ cells	Ink pencil (Sharpie)

**Table 2:** Morphometry of Schwann cell extensions.

Protocol	No. Cell Extensions	Median (25%; 75%) (μm)	Sum (μm)	Min. (μm)	Max. (μm)
Control 50	12	14.1 (11.0; 24.3)	221.4	3.3	61.8
Control 100	40	19.2 (8.2; 34.7)	979.5	4.3	79.9
Graphite 50	42	42.5 (15.2; 60.8)	2236.5	5.2	267.3
Graphite 100	102	19.4 (13.5; 29.5)	2420.1	4.8	120
Ink 50	16	21.4 (17.3; 28.4)	348.6	9.7	33.6
Ink 100	24	55.4 (29.0; 77.5)	1472.5	9.2	175.6

## Data Availability

The original contributions presented in this study are included in the article. Further inquiries can be directed to the corresponding author.

## Abbreviations

The following abbreviations are used in this manuscript:

SCs	Schwann cells
LV-SEM	Low-vacuum scanning electron microscopy
VP-SEM	Variable-pressure scanning electron microscopy
PHB	Polyhydroxybutyrate
NRG1	Neuregulin 1
NGF	Nerve growth factor
